# Supplementary material for: Comparative genomics provides new insights into the diversity, physiology, and sexuality of the only industrially exploited tremellomycete: Phaffia rhodozyma
Source: BMC Genomics. 2016 Nov 9;17:901. doi: 10.1186/s12864-016-3244-7 (PMC5103461; doi:10.1186/s12864-016-3244-7)
Supplement: Additional file 6: — List of orphan genes with links to PFAM (related to Additional file 1: Table S1). (ZIP 1428 kb) [file 12864_2016_3244_MOESM6_ESM.zip › PFAM_UNIQUE/G05590_P.html]

#                                                                            --- full sequence --- -------------- this domain -------------   hmm coord   ali coord   env coord
  
# target name        accession   tlen query name           accession   qlen   E-value  score  bias   #  of  c-Evalue  i-Evalue  score  bias  from    to  from    to  from    to  acc description of target
  
#------------------- ---------- ----- -------------------- ---------- ----- --------- ------ ----- --- --- --------- --------- ------ ----- ----- ----- ----- ----- ----- ----- ---- ---------------------
  
G05590\_P             -            106 AAA\_13               PF13166.1    712     0.024   11.8  10.0   1   1   0.00017     0.026   11.6  10.0   272   351     5    83     1    98 0.64 - AAA domain  
G05590\_P             -            106 AAA\_27               PF13514.1   1111      0.73    6.1   8.5   1   1    0.0037      0.92    5.7   8.5   857   934    16    92     3    99 0.63 - AAA domain  
G05590\_P             -            106 AF0941-like          PF14591.1    127       1.6    7.5   5.6   1   1    0.0008       2.4    6.9   5.5    60   106    12    60     7    81 0.83 - AF0941-like  
G05590\_P             -            106 AIP3                 PF03915.8    424       3.9    4.9   8.4   1   1      0.01       5.7    4.4   8.4    77   136     9    69     2   100 0.42 - Actin interacting protein 3  
G05590\_P             -            106 ALIX\_LYPXL\_bnd       PF13949.1    296      0.59    7.7   8.0   1   1    0.0016      0.74    7.4   8.0   109   172    14    92     2    99 0.62 - ALIX V-shaped domain binding to HIV  
G05590\_P             -            106 Alk\_phosphatase      PF00245.15   421     0.041   11.2   1.0   1   1     3e-05     0.045   11.1   1.0   281   356    18    96     3   101 0.83 - Alkaline phosphatase  
G05590\_P             -            106 Angiomotin\_C         PF12240.3    205      0.68    8.1   6.5   1   1   0.00047      0.94    7.6   6.5    98   169    27    94     3   101 0.82 - Angiomotin C terminal  
G05590\_P             -            106 APG6                 PF04111.7    315         2    6.1  12.0   1   1     0.014       2.5    5.7  12.0    12    93     9    90     2    98 0.53 - Autophagy protein Apg6  
G05590\_P             -            106 ApoLp-III            PF07464.6    155      0.14   10.9  11.0   1   1   0.00012      0.17   10.5  11.0    25   102     4    78     1    97 0.83 - Apolipophorin-III precursor (apoLp-III)  
G05590\_P             -            106 ASD2                 PF08687.6    264       5.5    4.9   8.4   1   1     0.011       6.3    4.7   8.4    43   102    17    91     1    98 0.56 - Apx/Shroom domain ASD2  
G05590\_P             -            106 Atg14                PF10186.4    302     0.011   13.3  10.5   1   1   5.8e-05     0.013   13.1  10.5    26   103    16    94     3    99 0.78 - UV radiation resistance protein and autophagy-related subunit 14  
G05590\_P             -            106 ATG16                PF08614.6    194       4.7    5.7  12.1   1   1     0.078        19    3.6  12.1    53   143    19    88     5    96 0.42 - Autophagy protein 16 (ATG16)  
G05590\_P             -            106 Bacillus\_HBL         PF05791.6    184       1.1    7.2   6.2   1   1    0.0014         1    7.4   5.4   115   180     4    71     1    75 0.83 - Bacillus haemolytic enterotoxin (HBL)  
G05590\_P             -            106 BicD                 PF09730.4    717       5.1    4.0   9.6   1   1     0.026       5.9    3.8   9.6   214   291    16    90     1    97 0.41 - Microtubule-associated protein Bicaudal-D  
G05590\_P             -            106 Borrelia\_P83         PF05262.6    489       5.1    3.9  12.7   1   1     0.016       5.8    3.7  12.7   201   288     2    89     1    99 0.48 - Borrelia P83/100 protein  
G05590\_P             -            106 Casc1                PF12366.3    244       2.3    6.3   7.1   1   1    0.0018       2.6    6.1   7.1    59   125    13    81     5    96 0.66 - Cancer susceptibility candidate 1  
G05590\_P             -            106 Cast                 PF10174.4    775       7.2    3.2  12.4   1   1     0.039       8.4    2.9  12.4   521   603    11    94     1    98 0.66 - RIM-binding protein of the cytomatrix active zone  
G05590\_P             -            106 CDC37\_N              PF03234.9    177       7.4    5.5   9.8   1   1     0.012        10    5.1   9.8    49   125     5    82     1    99 0.53 - Cdc37 N terminal kinase binding  
G05590\_P             -            106 CENP-F\_leu\_zip       PF10473.4    140       2.7    6.5  14.8   1   2     0.014       2.8    6.5   9.6    82   125     4    48     1    64 0.66 - Leucine-rich repeats of kinetochore protein Cenp-F/LEK1  
G05590\_P             -            106 CENP-F\_leu\_zip       PF10473.4    140       2.7    6.5  14.8   2   2      0.21        41    2.7   4.7    41   100    43    88    34    97 0.37 - Leucine-rich repeats of kinetochore protein Cenp-F/LEK1  
G05590\_P             -            106 CENP-F\_N             PF10481.4    307       3.3    5.7   7.1   1   1    0.0039       3.9    5.4   7.1   106   191    10    93     3   103 0.73 - Cenp-F N-terminal domain  
G05590\_P             -            106 Cgr1                 PF03879.9    109       3.9    6.4  11.8   1   1    0.0076       6.5    5.7  11.7    34    85    13    69     2    82 0.66 - Cgr1 family  
G05590\_P             -            106 Cluap1               PF10234.4    267       0.7    7.7   9.6   1   1   0.00076       1.1    7.0   9.6    72   160    10    93     1    97 0.83 - Clusterin-associated protein-1  
G05590\_P             -            106 COG5                 PF10392.4    132       4.1    6.0   7.7   1   1     0.025        15    4.2   7.7    33   110    13    92     2    96 0.70 - Golgi transport complex subunit 5  
G05590\_P             -            106 Cytochrom\_C552       PF02335.10   435       3.7    4.8   5.9   1   1    0.0021       4.1    4.6   5.9   285   337     6    58     2    96 0.65 - Cytochrome c552  
G05590\_P             -            106 DGCR6                PF07324.6    196       7.9    4.6   9.4   1   1    0.0016       9.4    4.3   9.4    84   155     9    82     2    93 0.73 - DiGeorge syndrome critical region 6 (DGCR6) protein  
G05590\_P             -            106 Dict-STAT-coil       PF09267.5    114      0.67    8.9   4.4   1   1   0.00093       1.9    7.4   4.4    29   111     4    95     2    98 0.74 - Dictyostelium STAT, coiled coil  
G05590\_P             -            106 DUF1216              PF06746.6    138      0.17   10.3   7.5   1   1   4.5e-05      0.27    9.7   7.5    11    74     7    69     1    94 0.80 - Protein of unknown function (DUF1216)  
G05590\_P             -            106 DUF1319              PF07028.6    126       2.5    7.0   8.4   1   2    0.0044       4.4    6.2   2.7    48    78     4    35     1    44 0.80 - Protein of unknown function (DUF1319)  
G05590\_P             -            106 DUF1319              PF07028.6    126       2.5    7.0   8.4   2   2    0.0049       4.9    6.1   1.1    35    89    29    83    28    87 0.82 - Protein of unknown function (DUF1319)  
G05590\_P             -            106 DUF1340              PF07067.6    236      0.14    9.9   1.5   1   1   2.7e-05      0.16    9.7   1.5    54   131     6    85     2    93 0.85 - Protein of unknown function (DUF1340)  
G05590\_P             -            106 DUF1359              PF07097.6    102      0.16   10.7   1.7   1   2      0.15   4.5e+02   -0.4   0.1    10    31    18    39     4    53 0.53 - Protein of unknown function (DUF1359)  
G05590\_P             -            106 DUF1359              PF07097.6    102      0.16   10.7   1.7   2   2   6.3e-05      0.19   10.5   0.4     8    46    53    91    43    97 0.86 - Protein of unknown function (DUF1359)  
G05590\_P             -            106 DUF1366              PF07104.6    116       8.4    4.7   7.2   1   1    0.0044        26    3.1   7.1    64   114    34    86     8    87 0.80 - Protein of unknown function (DUF1366)  
G05590\_P             -            106 DUF1451              PF07295.6    146       6.2    5.3   8.2   1   1     0.004         8    4.9   8.2     3    80     9    89     5    95 0.71 - Protein of unknown function (DUF1451)  
G05590\_P             -            106 DUF16                PF01519.11   102       5.2    6.0  10.7   1   1    0.0055        33    3.5  10.8     7    80     8    95     5    98 0.73 - Protein of unknown function DUF16  
G05590\_P             -            106 DUF1664              PF07889.7    126       6.8    5.2   8.7   1   1     0.031        11    4.5   8.7    46   110    16    82     1    96 0.44 - Protein of unknown function (DUF1664)  
G05590\_P             -            106 DUF1672              PF07901.6    277     0.079   10.6   5.3   1   1   1.6e-05     0.093   10.4   5.3   176   257     5    85     2    98 0.83 - Protein of unknown function (DUF1672)  
G05590\_P             -            106 DUF1754              PF08555.5     98       5.4    6.4   9.6   1   2   0.00072       1.4    8.2   6.0    47    93     8    54     2    59 0.78 - Eukaryotic family of unknown function (DUF1754)  
G05590\_P             -            106 DUF1754              PF08555.5     98       5.4    6.4   9.6   2   2      0.47   9.3e+02   -0.8   0.1    26    26    82    82    55    99 0.54 - Eukaryotic family of unknown function (DUF1754)  
G05590\_P             -            106 DUF1840              PF08895.6    105    0.0096   14.6   7.4   1   1   2.5e-06     0.015   14.0   7.1    28   102     8    82     4    84 0.78 - Domain of unknown function (DUF1840)  
G05590\_P             -            106 DUF2051              PF09738.4    302       1.2    7.0   8.2   1   1    0.0014       1.4    6.8   8.2   122   197    13    91     3    98 0.73 - Double stranded RNA binding protein (DUF2051)  
G05590\_P             -            106 DUF2130              PF09903.4    267      0.55    7.6   9.5   1   1   0.00032      0.64    7.4   8.8   186   245     1    66     1    86 0.82 - Uncharacterized protein conserved in bacteria (DUF2130)  
G05590\_P             -            106 DUF2203              PF09969.4    120       4.3    6.3   6.6   1   1    0.0014       8.6    5.3   6.6    23    77    13    74     1    98 0.49 - Uncharacterized conserved protein (DUF2203)  
G05590\_P             -            106 DUF2317              PF10079.4    542       7.5    3.7   7.0   1   1    0.0053       7.9    3.6   7.0   427   491     3    67     1    94 0.78 - Uncharacterized protein conserved in bacteria (DUF2317)  
G05590\_P             -            106 DUF2353              PF09789.4    319         2    6.2   9.3   1   1    0.0051       2.5    5.9   9.3    76   155    14    93     2    97 0.63 - Uncharacterized coiled-coil protein (DUF2353)  
G05590\_P             -            106 DUF2570              PF10828.3    110       9.8    4.5  11.1   1   2    0.0014       4.2    5.7   6.2    34    83    12    63     2    66 0.74 - Protein of unknown function (DUF2570)  
G05590\_P             -            106 DUF2570              PF10828.3    110       9.8    4.5  11.1   2   2     0.028        84    1.5   0.2    28    49    71    92    64    96 0.60 - Protein of unknown function (DUF2570)  
G05590\_P             -            106 DUF3053              PF11254.3    229      0.83    7.5   4.4   1   1   0.00013       0.8    7.6   3.6    75   137    30    95    22    98 0.75 - Protein of unknown function (DUF3053)  
G05590\_P             -            106 DUF3138              PF11336.3    514      0.11    9.3   3.6   1   1   4.1e-05      0.12    9.1   3.6    28   108    13    93     4    99 0.86 - Protein of unknown function (DUF3138)  
G05590\_P             -            106 DUF342               PF03961.8    451       5.3    4.0  11.4   1   1     0.011       5.7    3.9  11.4   333   409     5    81     1    96 0.78 - Protein of unknown function (DUF342)  
G05590\_P             -            106 DUF3450              PF11932.3    252       8.8    4.2   9.3   1   1    0.0094        11    3.8   9.3    22    94    20    91     5    97 0.76 - Protein of unknown function (DUF3450)  
G05590\_P             -            106 DUF3560              PF12083.3    126      0.23   10.2   6.1   1   1   9.8e-05      0.29    9.8   6.1    57   114    20    81     4    96 0.74 - Domain of unknown function (DUF3560)  
G05590\_P             -            106 DUF3584              PF12128.3   1201       5.2    2.9  12.1   1   1     0.045       6.2    2.6  12.1   679   756    11    88     2    99 0.29 - Protein of unknown function (DUF3584)  
G05590\_P             -            106 DUF3585              PF12130.3    142       2.1    6.9   7.4   1   2    0.0068        20    3.7   1.1    13    38    14    39     3    52 0.69 - Protein of unknown function (DUF3585)  
G05590\_P             -            106 DUF3585              PF12130.3    142       2.1    6.9   7.4   2   2   0.00074       2.2    6.8   3.5    71   131    31    92    28    99 0.79 - Protein of unknown function (DUF3585)  
G05590\_P             -            106 DUF3635              PF12330.3     98      0.35    9.7   6.5   1   2   0.00047       2.8    6.7   1.4    53    93     5    44     1    49 0.80 - Domain of unknown function (DUF3635)  
G05590\_P             -            106 DUF3635              PF12330.3     98      0.35    9.7   6.5   2   2   0.00052       3.1    6.6   0.5    50    92    52    94    41    99 0.64 - Domain of unknown function (DUF3635)  
G05590\_P             -            106 DUF4132              PF13569.1    180      0.59    8.3   6.6   1   2   0.00029      0.87    7.8   6.2     4    38     4    38     2    85 0.84 - Domain of unknown function (DUF4132)  
G05590\_P             -            106 DUF4132              PF13569.1    180      0.59    8.3   6.6   2   2      0.15   4.6e+02   -1.1   0.1    16    40    60    84    46    96 0.56 - Domain of unknown function (DUF4132)  
G05590\_P             -            106 DUF4140              PF13600.1    104       9.9    5.4   9.1   1   2     0.018         7    5.8   2.6    65    92    12    39     2    51 0.68 - N-terminal domain of unknown function (DUF4140)  
G05590\_P             -            106 DUF4140              PF13600.1    104       9.9    5.4   9.1   2   2     0.056        22    4.2   2.2    47   100    38    91    33    96 0.58 - N-terminal domain of unknown function (DUF4140)  
G05590\_P             -            106 DUF4164              PF13747.1     89       2.1    7.2   7.2   1   1     0.014        11    4.9   6.8     4    67    20    89    10    93 0.61 - Domain of unknown function (DUF4164)  
G05590\_P             -            106 DUF4175              PF13779.1    820      0.17    8.2   4.6   1   1   0.00052      0.18    8.1   4.6   657   717     6    64     2    98 0.85 - Domain of unknown function (DUF4175)  
G05590\_P             -            106 DUF4200              PF13863.1    126         3    6.5  14.1   1   1     0.019         6    5.5  14.1    22   108     3    88     1    92 0.85 - Domain of unknown function (DUF4200)  
G05590\_P             -            106 DUF4201              PF13870.1    177       5.7    5.0  11.3   1   2     0.008       2.8    6.0   1.6   155   176    13    34     1    35 0.73 - Domain of unknown function (DUF4201)  
G05590\_P             -            106 DUF4201              PF13870.1    177       5.7    5.0  11.3   2   2      0.19        67    1.5  10.4    51   158    15    81     9    99 0.41 - Domain of unknown function (DUF4201)  
G05590\_P             -            106 DUF4226              PF10774.4    112       1.8    7.3   5.8   1   2    0.0019        12    4.7   0.7    40    69    13    43     4    48 0.82 - Domain of unknown function (DUF4226)  
G05590\_P             -            106 DUF4226              PF10774.4    112       1.8    7.3   5.8   2   2    0.0019        12    4.7   0.5     8    34    54    80    47    96 0.73 - Domain of unknown function (DUF4226)  
G05590\_P             -            106 DUF4398              PF14346.1    103    0.0082   14.9   9.6   1   1     1e-05     0.012   14.3   9.6    15    91    18    91     7    96 0.78 - Domain of unknown function (DUF4398)  
G05590\_P             -            106 DUF4407              PF14362.1    301       1.8    6.1   8.7   1   1     0.016       5.9    4.4   8.7   131   171    35    97     2   104 0.66 - Domain of unknown function (DUF4407)  
G05590\_P             -            106 DUF4455              PF14643.1    474       2.7    5.2   8.1   1   1    0.0011       3.4    4.8   8.1    16    99    18    95     4    98 0.73 - Domain of unknown function (DUF4455)  
G05590\_P             -            106 DUF4559              PF15112.1    307       4.1    5.1   6.2   1   1    0.0049       4.9    4.9   6.2   255   304    12    63     1    96 0.72 - Domain of unknown function (DUF4559)  
G05590\_P             -            106 DUF465               PF04325.8     49         1    7.8   9.1   1   3       0.1   1.5e+02    0.8   0.1    10    21     6    17     5    21 0.81 - Protein of unknown function (DUF465)  
G05590\_P             -            106 DUF465               PF04325.8     49         1    7.8   9.1   2   3   0.00023      0.34    9.3   3.4     3    24    21    43    19    55 0.75 - Protein of unknown function (DUF465)  
G05590\_P             -            106 DUF465               PF04325.8     49         1    7.8   9.1   3   3       0.4     6e+02   -1.1   0.0     9    17    71    79    59    87 0.71 - Protein of unknown function (DUF465)  
G05590\_P             -            106 DUF724               PF05266.9    190     0.099   10.9  12.0   1   1   0.00013      0.13   10.6  12.0   110   180     2    82     1    92 0.72 - Protein of unknown function (DUF724)  
G05590\_P             -            106 DUF745               PF05335.8    188       3.8    5.7  11.4   1   1     0.015        11    4.1  11.5    66   136    16    88     3    96 0.47 - Protein of unknown function (DUF745)  
G05590\_P             -            106 DUF812               PF05667.6    594       4.2    4.4  11.6   1   1     0.021       5.4    4.0  11.6   302   381    13    93     1    98 0.37 - Protein of unknown function (DUF812)  
G05590\_P             -            106 DUF869               PF05911.6    769       6.7    3.5  11.1   1   1     0.031       8.1    3.2  11.1    55   130    16    92     2    99 0.57 - Plant protein of unknown function (DUF869)  
G05590\_P             -            106 DUF883               PF05957.8     94       4.7    6.4   8.3   1   1     0.017       7.8    5.7   7.6    12    71     5    66     1    80 0.82 - Bacterial protein of unknown function (DUF883)  
G05590\_P             -            106 DUF964               PF06133.6    108       2.4    6.9   9.8   1   1    0.0012       1.5    7.6   7.5    23    78     5    60     1    73 0.85 - Protein of unknown function (DUF964)  
G05590\_P             -            106 Dynamitin            PF04912.9    388       6.9    4.0   7.7   1   2    0.0039       3.9    4.8   1.7   201   239     9    46     2    52 0.78 - Dynamitin  
G05590\_P             -            106 Dynamitin            PF04912.9    388       6.9    4.0   7.7   2   2     0.024        24    2.2   0.8   333   367    58    92    47    97 0.53 - Dynamitin  
G05590\_P             -            106 E2R135               PF11570.3    136       9.5    5.0   7.4   1   1     0.011        21    3.8   7.4     1    66     6    72     6    98 0.70 - Coiled-coil receptor-binding R-domain of colicin E2  
G05590\_P             -            106 End3                 PF12761.2    195       8.9    4.6  11.0   1   1      0.01        10    4.3  11.0   104   193     3    87     1    95 0.72 - Actin cytoskeleton-regulatory complex protein END3  
G05590\_P             -            106 Endonuc\_Holl         PF10107.4    156      0.59    8.5   7.8   1   1   0.00032      0.64    8.4   7.4    13    73     4    63     1    71 0.86 - Endonuclease related to archaeal Holliday junction resolvase  
G05590\_P             -            106 ERM                  PF00769.14   246      0.62    8.2  14.6   1   1     0.005      0.72    8.0  14.6    12   103     2    96     1    98 0.91 - Ezrin/radixin/moesin family  
G05590\_P             -            106 Exonuc\_VII\_L         PF02601.10   319       3.7    5.3   9.1   1   1    0.0088       4.1    5.2   9.1   152   226     4    88     1    99 0.46 - Exonuclease VII, large subunit  
G05590\_P             -            106 EzrA                 PF06160.7    560       7.7    3.2  10.8   1   1     0.022        14    2.3  10.8    57   129    14    89     1    97 0.32 - Septation ring formation regulator, EzrA  
G05590\_P             -            106 FAM91\_C              PF14648.1    450         3    5.2   5.9   1   1   0.00058       3.4    5.0   5.9   251   341     9    94     3    99 0.75 - FAM91 C-terminus  
G05590\_P             -            106 FemAB                PF02388.11   406       2.3    5.4   8.0   1   1    0.0023       2.7    5.2   8.0   243   302     7    76     2    93 0.71 - FemAB family  
G05590\_P             -            106 FH2                  PF02181.18   370       1.7    6.1   8.3   1   1    0.0023       2.3    5.7   8.3   265   342     4    73     1    96 0.61 - Formin Homology 2 Domain  
G05590\_P             -            106 Fib\_alpha            PF08702.5    146      0.56    9.0   9.9   1   1    0.0021      0.72    8.6   9.9    37   131     3    95     1    97 0.73 - Fibrinogen alpha/beta chain family  
G05590\_P             -            106 Filament             PF00038.16   312       1.7    6.7   8.6   1   1     0.013       2.1    6.4   8.6    20   252    19    83     1    97 0.37 - Intermediate filament protein  
G05590\_P             -            106 FlaF                 PF07309.6    113      0.28    9.8   2.7   1   1   9.5e-05      0.57    8.8   2.7    15    58    33    73     3    97 0.75 - Flagellar protein FlaF  
G05590\_P             -            106 GBP\_C                PF02841.9    297       1.9    6.2  13.2   1   1    0.0025       2.2    6.1  13.2   185   266    12    90     4    99 0.79 - Guanylate-binding protein, C-terminal domain  
G05590\_P             -            106 GCOM2                PF15328.1    223       3.2    6.1   9.0   1   1    0.0076       3.8    5.8   9.0    82   173     5    91     1   102 0.53 - Putative GRINL1B complex locus protein 2  
G05590\_P             -            106 GRIN\_C               PF15235.1    137       2.8    6.7   8.3   1   2   0.00066         2    7.2   6.3    74   122     5    59     4    66 0.70 - G protein-regulated inducer of neurite outgrowth C-terminus  
G05590\_P             -            106 GRIN\_C               PF15235.1    137       2.8    6.7   8.3   2   2     0.068     2e+02    0.7   0.4    75    98    44    67    39    98 0.50 - G protein-regulated inducer of neurite outgrowth C-terminus  
G05590\_P             -            106 HCR                  PF07111.7    739      0.95    6.0   6.6   1   1    0.0014         1    5.9   6.6   168   231    16    81     2    99 0.81 - Alpha helical coiled-coil rod protein (HCR)  
G05590\_P             -            106 HemX                 PF04375.9    372       1.9    5.6   5.8   1   1    0.0023       2.3    5.4   5.8    96   165     5    79     1    96 0.71 - HemX  
G05590\_P             -            106 HGTP\_anticodon2      PF12745.2    273       7.3    4.3   8.3   1   1    0.0039       5.9    4.6   7.2   124   194     7    73     1    79 0.64 - Anticodon binding domain of tRNAs  
G05590\_P             -            106 HOOK                 PF05622.7    713       1.8    4.9  13.8   1   1    0.0077       2.1    4.7  13.8   503   589    11    95     2    98 0.88 - HOOK protein  
G05590\_P             -            106 HrpE                 PF06188.7    191      0.49    8.8   3.2   1   1    0.0002       0.6    8.5   3.2    78   129    27    79     3    97 0.70 - HrpE/YscL/FliH and V-type ATPase subunit E  
G05590\_P             -            106 IFT57                PF10498.4    359       1.2    6.4  10.5   1   1     0.003       1.5    6.1  10.5   226   308     5    89     1    97 0.74 - Intra-flagellar transport protein 57  
G05590\_P             -            106 IncA                 PF04156.9    191     0.053   11.8  11.7   1   1   0.00076     0.074   11.3  11.7    87   178     9    95     2    97 0.75 - IncA protein  
G05590\_P             -            106 Kinetocho\_Slk19      PF12709.2     87       1.9    7.3  11.0   1   1     0.032        38    3.1  10.8     3    86     8    87     6    88 0.62 - Central kinetochore-associated  
G05590\_P             -            106 LMP                  PF04778.7    157      0.18   10.2   9.8   1   1   0.00012      0.25    9.8   9.8    63   153    11    96     2    99 0.78 - LMP repeated region  
G05590\_P             -            106 LXG                  PF04740.7    204       1.1    7.7   9.7   1   1    0.0018       1.8    7.0   9.7    71   155    13    95     4    97 0.88 - LXG domain of WXG superfamily  
G05590\_P             -            106 MAT1                 PF06391.8    200       7.8    4.7  11.5   1   1     0.011        10    4.3  10.4   121   176     9    63     1    88 0.57 - CDK-activating kinase assembly factor MAT1  
G05590\_P             -            106 MCPsignal            PF00015.16   213      0.44    8.7   6.7   1   1    0.0013       0.8    7.9   6.7    88   161    19    94     9    98 0.65 - Methyl-accepting chemotaxis protein (MCP) signalling domain  
G05590\_P             -            106 MCRS\_N               PF13325.1    199         2    6.6   5.1   1   1     0.001         3    6.0   5.1   137   198    10    70     3    71 0.75 - N-terminal region of micro-spherule protein  
G05590\_P             -            106 Med11                PF10280.4    117     0.069   11.9   3.5   1   1   5.9e-05      0.12   11.1   3.5    39    86     4    63     2    99 0.70 - Mediator complex protein  
G05590\_P             -            106 Med28                PF11594.3    106       2.2    7.2   5.5   1   1    0.0013       7.7    5.5   5.5    37    83    49    95     2   102 0.77 - Mediator complex subunit 28  
G05590\_P             -            106 Med9                 PF07544.8     83       1.8    7.1   8.4   1   2     0.016        12    4.4   1.4     8    38     4    34     1    42 0.74 - RNA polymerase II transcription mediator complex subunit 9  
G05590\_P             -            106 Med9                 PF07544.8     83       1.8    7.1   8.4   2   2    0.0033       2.5    6.6   0.8    35    80    47    89    42    92 0.82 - RNA polymerase II transcription mediator complex subunit 9  
G05590\_P             -            106 Myosin\_tail\_1        PF01576.14   859      0.72    6.2  12.8   1   1    0.0056      0.84    5.9  12.8   552   634     8    93     1    98 0.40 - Myosin tail  
G05590\_P             -            106 Nop25                PF09805.4    137       2.8    6.7   9.6   1   1    0.0093       3.7    6.3   9.6    46   124     8    88     2    99 0.59 - Nucleolar protein 12 (25kDa)  
G05590\_P             -            106 P4Ha\_N               PF08336.6    134       9.1    4.8   7.1   1   1      0.02        40    2.7   7.1     6    45     1    40     1    99 0.79 - Prolyl 4-Hydroxylase alpha-subunit, N-terminal region  
G05590\_P             -            106 PcfJ                 PF14284.1    169     0.046   11.9   7.6   1   1   8.9e-06     0.053   11.7   7.6     8    82     3    75     1    95 0.82 - PcfJ-like protein  
G05590\_P             -            106 PEP-utilisers\_N      PF05524.8    123      0.44    9.0   6.5   1   1   0.00037      0.55    8.7   6.5    31    98    13    85     2    96 0.68 - PEP-utilising enzyme, N-terminal  
G05590\_P             -            106 Peptidase\_S46        PF10459.4    698       2.7    4.9   6.0   1   1    0.0034       2.9    4.8   6.0   291   376     8    93     1   100 0.63 - Peptidase S46  
G05590\_P             -            106 Pox\_A11              PF05061.8    314      0.69    7.6   4.0   1   1   0.00025      0.76    7.4   4.0    36   133     1    93     1   102 0.66 - Poxvirus A11 Protein  
G05590\_P             -            106 Prominin             PF05478.6    807     0.031   10.5   4.0   1   1     5e-05     0.033   10.4   4.0   198   295     5    94     1   104 0.79 - Prominin  
G05590\_P             -            106 PspA\_IM30            PF04012.7    221       9.9    4.1  13.8   1   1     0.051        19    3.2  13.8    54   143     5    92     1    96 0.57 - PspA/IM30 family  
G05590\_P             -            106 Rabaptin             PF03528.10   106         9    5.0  11.3   1   2     0.079        52    2.5   2.8    66    90    16    38     1    49 0.54 - Rabaptin  
G05590\_P             -            106 Rabaptin             PF03528.10   106         9    5.0  11.3   2   2    0.0065       4.3    6.0   3.3    58   101    49    93    36    97 0.84 - Rabaptin  
G05590\_P             -            106 Scaffolding\_pro      PF11418.3     97       0.5    9.4   3.4   1   1   0.00028      0.83    8.7   3.3    40    80    37    77     3    91 0.74 - Phi29 scaffolding protein  
G05590\_P             -            106 Sec2p                PF06428.6    101      0.62    8.6  13.0   1   1    0.0017       1.5    7.4  13.0     6    91     8    94     2    99 0.84 - GDP/GTP exchange factor Sec2p  
G05590\_P             -            106 Sec8\_exocyst         PF04048.9    142      0.06   11.7   9.3   1   1   8.6e-05     0.074   11.4   9.3    47   133    10    94     1    98 0.80 - Sec8 exocyst complex component specific domain  
G05590\_P             -            106 SF-assemblin         PF06705.6    247       4.1    5.1  10.0   1   1    0.0062       6.2    4.6  10.0    44    98     5    61     2    96 0.46 - SF-assemblin/beta giardin  
G05590\_P             -            106 SlyX                 PF04102.7     69      0.64    9.1   5.4   1   2      0.43   1.5e+02    1.5   1.0    20    50    19    35     3    62 0.51 - SlyX  
G05590\_P             -            106 SlyX                 PF04102.7     69      0.64    9.1   5.4   2   2     0.001      0.36    9.9   0.4    10    41    60    91    51    97 0.86 - SlyX  
G05590\_P             -            106 Snapin\_Pallidin      PF14712.1     92       3.1    6.9   9.4   1   1     0.013       6.4    5.8   8.9    12    91     7    88     3    89 0.63 - Snapin/Pallidin  
G05590\_P             -            106 Snf7                 PF03357.16   171       5.3    5.1  11.3   1   1     0.016       6.7    4.7  11.3     5    92     3    92     1    98 0.72 - Snf7  
G05590\_P             -            106 Spc7                 PF08317.6    326      0.19    9.0  14.6   1   1    0.0018      0.33    8.2  14.6   176   254     9    92     1    97 0.80 - Spc7 kinetochore protein  
G05590\_P             -            106 Spectrin             PF00435.16   105         1    8.4  10.6   1   2    0.0022       1.3    8.0   6.0    30    70    13    52     3    62 0.74 - Spectrin repeat  
G05590\_P             -            106 Spectrin             PF00435.16   105         1    8.4  10.6   2   2     0.073        44    3.1   1.6    35    74    48    90    40    97 0.53 - Spectrin repeat  
G05590\_P             -            106 Striatin             PF08232.7    135       8.1    5.4  11.2   1   2     0.017        11    4.9  10.4    54   127     3    79     1    89 0.51 - Striatin family  
G05590\_P             -            106 Striatin             PF08232.7    135       8.1    5.4  11.2   2   2      0.39   2.6e+02    0.6   0.0    25    46    75    96    64    98 0.68 - Striatin family  
G05590\_P             -            106 SWI-SNF\_Ssr4         PF08549.5    669     0.028   11.4   5.6   1   1     1e-05     0.031   11.2   5.6   366   433     8    75     3    95 0.84 - Fungal domain of unknown function (DUF1750)  
G05590\_P             -            106 Syntaxin-6\_N         PF09177.6     97       1.3    8.2   5.5   1   2    0.0029       1.9    7.6   4.4    39    94    10    64     1    81 0.66 - Syntaxin 6, N-terminal  
G05590\_P             -            106 Syntaxin-6\_N         PF09177.6     97       1.3    8.2   5.5   2   2      0.13        84    2.4   0.3    13    50    62    79    39    98 0.44 - Syntaxin 6, N-terminal  
G05590\_P             -            106 Syntaxin\_2           PF14523.1    102       6.5    5.5  10.0   1   2    0.0022       1.9    7.3   5.4    46    99    12    61     2    64 0.84 - Syntaxin-like protein  
G05590\_P             -            106 Syntaxin\_2           PF14523.1    102       6.5    5.5  10.0   2   2       0.2   1.7e+02    0.9   0.2    12    31    70    89    61    98 0.37 - Syntaxin-like protein  
G05590\_P             -            106 TACC                 PF05010.9    207      0.83    8.0   9.9   1   2      0.11        98    1.3   2.6     9    35     6    32     1    45 0.39 - Transforming acidic coiled-coil-containing protein (TACC)  
G05590\_P             -            106 TACC                 PF05010.9    207      0.83    8.0   9.9   2   2   7.4e-05     0.063   11.7   0.8     7    51    45    89    42    95 0.92 - Transforming acidic coiled-coil-containing protein (TACC)  
G05590\_P             -            106 TarH                 PF02203.10   171      0.45    9.0   3.0   1   1    0.0002       0.4    9.2   1.9   111   161     8    57     2    68 0.87 - Tar ligand binding domain homologue  
G05590\_P             -            106 Tektin               PF03148.9    384       7.8    3.6  10.3   1   1     0.023        14    2.7  10.0   221   293     5    82     1    89 0.75 - Tektin family  
G05590\_P             -            106 TEX13                PF15186.1    152       1.4    7.2   7.1   1   2   7.4e-05      0.45    8.8   3.5   106   141     2    38     1    54 0.82 - Testis-expressed sequence 13 protein family  
G05590\_P             -            106 TEX13                PF15186.1    152       1.4    7.2   7.1   2   2     0.031   1.8e+02    0.3   0.4    87    87    63    63    35    96 0.47 - Testis-expressed sequence 13 protein family  
G05590\_P             -            106 TIP120               PF08623.5    169       1.4    7.2   5.6   1   1    0.0014       1.6    6.9   5.6    89   150     7    71     3    94 0.78 - TATA-binding protein interacting (TIP20)  
G05590\_P             -            106 TMCO5                PF14992.1    280       1.7    6.4  11.0   1   1    0.0038       2.3    6.0  11.0    55   135     9    87     2    98 0.44 - TMCO5 family  
G05590\_P             -            106 TMF\_DNA\_bd           PF12329.3     74       8.1    5.0  18.6   1   2      0.44   1.6e+02    0.9  11.8    21    54     4    40     1    72 0.67 - TATA element modulatory factor 1 DNA binding  
G05590\_P             -            106 TMF\_DNA\_bd           PF12329.3     74       8.1    5.0  18.6   2   2     0.034        12    4.5   4.0    37    71    58    92    42    95 0.72 - TATA element modulatory factor 1 DNA binding  
G05590\_P             -            106 TMPIT                PF07851.8    330       1.4    6.6   9.0   1   1    0.0014       1.7    6.3   9.0    11    87     9    87     1    97 0.40 - TMPIT-like protein  
G05590\_P             -            106 Tox-ODYAM1           PF15642.1    385       8.3    3.6   7.3   1   1    0.0015       9.2    3.5   7.3    90   162     3    82     1    97 0.52 - Toxin in Odyssella and Amoebophilus  
G05590\_P             -            106 TPR\_MLP1\_2           PF07926.7    132       7.9    4.9  15.3   1   1    0.0086         2    6.8   6.3    11    67    36    93    31    97 0.76 - TPR/MLP1/MLP2-like protein  
G05590\_P             -            106 Transposase\_22       PF02994.9    370         6    3.9   8.8   1   1    0.0097       7.2    3.6   8.8    49   125     8    81     2    97 0.41 - L1 transposable element  
G05590\_P             -            106 Tropomyosin          PF00261.15   237       1.3    6.7  12.8   1   1    0.0096       2.3    5.9  12.5     3    85    15    96     9   101 0.79 - Tropomyosin  
G05590\_P             -            106 Tropomyosin\_1        PF12718.2    143       7.9    5.0  15.5   1   1    0.0043       1.4    7.4   8.1     4    69    32    95    30    98 0.85 - Tropomyosin like  
G05590\_P             -            106 Use1                 PF09753.4    251       4.4    5.3   6.8   1   1    0.0069       5.1    5.1   6.8    40   111     4    75     1    98 0.76 - Membrane fusion protein Use1  
G05590\_P             -            106 V-SNARE              PF05008.10    79         2    7.3   9.1   1   2     0.089        66    2.4   6.7    28    77    13    63     1    65 0.61 - Vesicle transport v-SNARE protein N-terminus  
G05590\_P             -            106 V-SNARE              PF05008.10    79         2    7.3   9.1   2   2    0.0029       2.2    7.2   0.9    21    49    50    78    35    94 0.83 - Vesicle transport v-SNARE protein N-terminus  
G05590\_P             -            106 Vps51                PF08700.6     87       2.4    6.8   6.7   1   1     0.061        61    2.2   6.7    17    40    15    38     2    92 0.67 - Vps51/Vps67  
G05590\_P             -            106 V\_ATPase\_I           PF01496.14   759       1.8    5.0   8.0   1   1    0.0069       2.1    4.8   8.0    34   115    13    95     2   100 0.70 - V-type ATPase 116kDa subunit family  
G05590\_P             -            106 WEMBL                PF05701.6    522       9.6    3.2  15.4   1   1     0.069        15    2.6  15.4   255   330     8    89     1    98 0.35 - Weak chloroplast movement under blue light  
G05590\_P             -            106 WTX                  PF09422.5    471       1.8    6.1   5.9   1   1   0.00034         2    5.9   5.9   125   213     4    90     1   101 0.79 - WTX protein  
G05590\_P             -            106 YkyA                 PF10368.4    204      0.94    7.4  11.8   1   1    0.0016       1.4    6.9  11.8    66   137    11    90     2    95 0.66 - Putative cell-wall binding lipoprotein  
G05590\_P             -            106 YlqD                 PF11068.3    131       3.1    6.5   9.9   1   1    0.0069       4.1    6.1   9.9    20    98     6    80     3    94 0.72 - YlqD protein  
G05590\_P             -            106 zf-C4H2              PF10146.4    230       6.9    5.3  11.5   1   1     0.028        13    4.4  11.5    27   115     4    95     1   101 0.56 - Zinc finger-containing protein  
G05590\_P             -            106 zf-CpG\_bind\_C        PF12269.3    236       2.5    6.2   7.8   1   2     0.001         3    5.9   6.8    30    82     6    57     2    81 0.73 - CpG binding protein zinc finger C terminal domain  
G05590\_P             -            106 zf-CpG\_bind\_C        PF12269.3    236       2.5    6.2   7.8   2   2     0.028        83    1.2   0.6    27    68    52    93    43    98 0.52 - CpG binding protein zinc finger C terminal domain  
G05590\_P             -            106 Zw10                 PF06248.8    593       3.3    4.4   7.0   1   1    0.0036       3.6    4.3   7.0    69   157     6    89     1    97 0.77 - Centromere/kinetochore Zw10
